# 2,2,6,6-Tetra­kis(biphenyl-2-yl)-4,4,8,8-tetra­methyl­cyclo­tetra­siloxane

**DOI:** 10.1107/S1600536809031961

**Published:** 2009-08-19

**Authors:** Erik P. A. Couzijn, Martin Lutz, Anthony L. Spek, Koop Lammertsma

**Affiliations:** a Department of Chemistry and Pharmaceutical Sciences, Faculty of Sciences, VU University Amsterdam, De Boelelaan 1083, 1081 HV Amsterdam, The Netherlands; b Bijvoet Center for Biomolecular Research, Crystal and Structural Chemistry, Faculty of Science, Utrecht University, Padualaan 8, 3584 CH Utrecht, The Netherlands

## Abstract

The title compound, [–Si(C_12_H_9_)_2_OSi(CH_3_)_2_O–]_2_, was obtained unintentionally as the product of an attempted crystallization of caesium bis­(biphenyl-2,2′-di­yl)fluoro­silicate from dimethyl­formamide. In the crystal, the mol­ecule is located on an inversion center and the siloxane ring adopts a twist-chair conformation with the two dimethyl-substituted Si atoms lying 0.7081 (5) Å out of the plane defined by the two bis­(biphenyl-2-yl)-substituted Si atoms and the four O atoms. In each Si(C_12_H_9_)_2_ unit, the orientation of one terminal phenyl ring relative to the phenyl­ene ring of the other biphenyl moiety suggests a parallel displaced π–π stacking inter­action [centroid distance = 4.2377 (11) Å and dihedral angle = 15.40 (9)°].

## Related literature

For general background to stable compounds of penta­valent, anionic silicon bearing five organic substituents, see: Couzijn *et al.* (2004 ▶, 2006 ▶, 2009 ▶); Deerenberg *et al.* (2002 ▶); de Keijzer *et al.* (1997 ▶). For related structures, see: Malinovskii *et al.* (2007 ▶); Steinfink *et al.* (1955 ▶); Hensen *et al.* (1997 ▶). For puckering analysis,, see: Evans & Boeyens (1989 ▶). Bis(biphenyl-2,2′-di­yl)silane was synthesized using a slight modification of a literature procedure (Gilman & Gorsich, 1958 ▶).
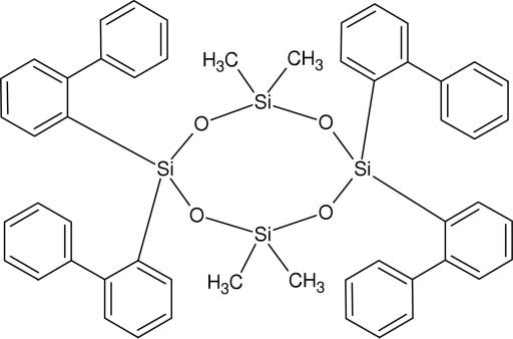


## Experimental

### 

#### Crystal data

C_52_H_48_O_4_Si_4_
*M* *_r_* = 849.26
Orthorhombic, 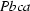

*a* = 17.3418 (2) Å
*b* = 14.6488 (2) Å
*c* = 17.9584 (2) Å
*V* = 4562.09 (10) Å^3^
*Z* = 4
Mo *K*α radiation
μ = 0.18 mm^−1^
*T* = 110 K
0.30 × 0.12 × 0.03 mm

#### Data collection

Nonius KappaCCD diffractometer
Absorption correction: none
68467 measured reflections
4317 independent reflections
3247 reflections with *I* > 2σ(*I*)
*R*_int_ = 0.081

#### Refinement

*R*[*F* ^2^ > 2σ(*F* ^2^)] = 0.036
*wR*(*F* ^2^) = 0.097
*S* = 1.07
4317 reflections
273 parameters
H-atom parameters constrained
Δρ_max_ = 0.26 e Å^−3^
Δρ_min_ = −0.33 e Å^−3^

### 

Data collection: *COLLECT* (Nonius, 1999 ▶); cell refinement: *HKL-2000* (Otwinowski & Minor, 1997 ▶); data reduction: *HKL-2000* and *SORTAV* (Blessing, 1997 ▶); program(s) used to solve structure: *SIR97* (Altomare *et al.*, 1999 ▶); program(s) used to refine structure: *SHELXL97* (Sheldrick, 2008 ▶); molecular graphics: *PLATON* (Spek, 2009 ▶); software used to prepare material for publication: manual editing of *SHELXL* output.

## Supplementary Material

Crystal structure: contains datablocks I, global. DOI: 10.1107/S1600536809031961/vm2002sup1.cif

Structure factors: contains datablocks I. DOI: 10.1107/S1600536809031961/vm2002Isup2.hkl

Additional supplementary materials:  crystallographic information; 3D view; checkCIF report

## Figures and Tables

**Table:** 

Si1—O1	1.6287 (12)
Si1—O2	1.6290 (13)
Si1—C12	1.8684 (19)
Si1—C11	1.8746 (17)
Si2—O2^i^	1.6342 (13)
Si2—O1	1.6347 (12)
Si2—C2	1.8452 (19)
Si2—C1	1.8494 (19)

**Table:** 

O1—Si1—O2	109.98 (7)
O2^i^—Si2—O1	107.88 (7)
Si1—O1—Si2	141.85 (8)
Si1—O2—Si2^i^	142.12 (8)

**Table:** 

O1—Si2—O2^i^—Si1^i^	−74.36 (14)
O2—Si1—O1—Si2	−52.51 (15)
O2^i^—Si2—O1—Si1	84.84 (14)
O1—Si1—O2—Si2^i^	−37.42 (15)
C11—C61—C71—C81	81.5 (2)
C12—C62—C72—C82	56.1 (3)

Symmetry code: (i) 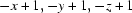.

**Fig. 1.**
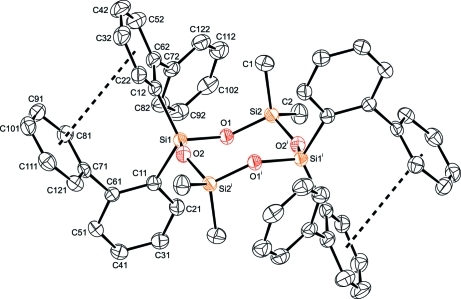
Displacement ellipsoid plot of [–Si(C12H9)2OSi(CH3)2O–]2 with ellipsoids drawn at the 50% probability level. The parallel displaced π-π stacking interaction is indicated by a dashed line between the ring centroids. Hydrogen atoms are omitted for clarity. Symmetry operation i: 1 - x, 1 - y, 1 - z.

## supplementary crystallographic information

### Comment

Our research is focused on stable compounds of pentavalent, anionic silicon bearing five organic substituents (Couzijn *et al.*, 2009; Couzijn *et al.*, 2006; Couzijn *et al.*, 2004). Such pentaorganosilicates are commonly proposed as intermediates for nucleophilic substitution reactions on silanes, but were only recently characterized in the condensed phase (*e.g.*, de Keijzer *et al.*, 1997; Deerenberg *et al.*, 2002). In solution, these five-coordinate species undergo intramolecular substituent interchange *via* Berry pseudorotation and related processes. For a better understanding of the influence of the substituents on the stereomutational barrier, we synthesized bis(biphenyl-2,2'-diyl)fluorosilicate as the caesium and tetramethylammonium salts (Couzijn *et al.*, 2009). While attempting to crystallize these salts, we obtained crystals of the title compound, [–Si(C_12_H_9_)_2_OSi(CH_3_)_2_O–]_2_, instead. This siloxane was most probably formed by reaction with silicone grease and adventitious water.

The title compound crystallizes with C_i_ point group symmetry, adopting a twist-chair conformation of the eight-membered siloxane ring (Fig. 1, Table 1). A ring puckering analysis (Evans & Boeyens, 1989) shows that the out-of-plane displacements in the ring can be described as a linear combination of the E_3g_ (*sin* form) and E_3g_ (*cos* form) normal modes, respectively, in a ratio of 0.891:0.109. The two bis(biphenyl)-substituted silicon atoms and the four oxygen atoms lie in a plane (RMS deviation 0.025 Å), whereas the two dimethyl-substituted silicon atoms are situated at 0.7081 (5)Å above and below this plane, respectively. Similar arrangements have been reported for octamethyl- (Steinfink *et al.*, 1955) and 2,2,6,6-tetraphenyl-4,4,8,8-tetramethyltetrasiloxane (Malinovskii *et al.*, 2007). Each Si(C_12_H_9_)_2_ unit features a parallel displaced π-π stacking interaction between one terminal phenyl ring and the phenylene ring of the other biphenyl moiety. The ring centroids are 4.2377 (11)Å apart and their vector makes an angle of 38.65° with the phenylene plane, while the ring planes make a dihedral angle of 15.40 (9)°.

### Experimental

General procedures: dimethylformamide (DMF) was distilled from phenylzinc iodide and stored in a glovebox on 3Å molecular sieves. Commercial caesium fluoride was dried *in vacuo* at > 373 K. Bis(biphenyl-2,2'-diyl)silane was synthesized from 1,1'-dibromobiphenyl and tetrachlorosilane using a slight modification of a literature procedure (Gilman & Gorsich, 1958).

The title compound was obtained as follows. In the purified nitrogen atmosphere of a glovebox, a flame-dried Schlenk tube was charged with caesium fluoride (71.45 mg, 470 µmol) and bis(biphenyl-2,2'-diyl)silane (73.95 mg, 222 µmol). Anhydrous DMF (3 ml) was added and the mixture was stirred in the glovebox for 2 days. ^19^F and ^1^H NMR indicated almost quantitative conversion to the desired caesium bis(biphenyl-2,2'-diyl)fluorosilicate (Couzijn *et al.*, 2009). The clear colorless supernate was filtered through glass wool in the glovebox and evaporated on a Schlenk line to afford the crude fluorosilicate as a white solid. Recrystallization of the fluorosilicate from DMF was initially performed by cooling the Schlenk tube in a freezer outside the glovebox. After repeated attempts, colorless blocks were obtained that were shown by X-ray analysis to be the title compound. Recrystallizations of the fluorosilicate using Young-type glassware (closed with a greaseless Teflon tap) in the freezer of a glovebox invariably afforded an amorphous solid.

### Refinement

All H atoms were located in difference Fourier maps and refined using a riding model (including free rotation of the methyl substituents), with C—H = 0.95–0.99 Å and with *U*_iso_(H) = 1.2 (1.5 for methyl groups) times *U*_eq_(C).

### Crystal data

**Table:**

C_52_H_48_O_4_Si_4_	*F*(000) = 1792
*M**_r_* = 849.26	*D*_x_ = 1.236 Mg m^−^^3^
Orthorhombic, *P**b**c**a*	Mo *K*α radiation, λ = 0.71073 Å
Hall symbol: -P 2ac 2ab	Cell parameters from 88729 reflections
*a* = 17.3418 (2) Å	θ = 1.0–25.7°
*b* = 14.6488 (2) Å	µ = 0.18 mm^−^^1^
*c* = 17.9584 (2) Å	*T* = 110 K
*V* = 4562.09 (10) Å^3^	Needle, colourless
*Z* = 4	0.30 × 0.12 × 0.03 mm

### Data collection

**Table:**

Nonius KappaCCD diffractometer	3247 reflections with *I* > 2σ(*I*)
Radiation source: rotating anode	*R*_int_ = 0.081
graphite	θ_max_ = 25.7°, θ_min_ = 2.1°
φ and ω scans	*h* = −21→21
68467 measured reflections	*k* = −17→17
4317 independent reflections	*l* = −21→21

### Refinement

**Table:**

Refinement on *F*^2^	Primary atom site location: structure-invariant direct methods
Least-squares matrix: full	Secondary atom site location: difference Fourier map
*R*[*F*^2^ > 2σ(*F*^2^)] = 0.036	Hydrogen site location: difference Fourier map
*wR*(*F*^2^) = 0.097	H-atom parameters constrained
*S* = 1.07	*w* = 1/[σ^2^(*F*_o_^2^) + (0.0428*P*)^2^ + 1.7858*P*] where *P* = (*F*_o_^2^ + 2*F*_c_^2^)/3
4317 reflections	(Δ/σ)_max_ = 0.001
273 parameters	Δρ_max_ = 0.26 e Å^−^^3^
0 restraints	Δρ_min_ = −0.33 e Å^−^^3^

### Special details

**Table:**

Geometry. All e.s.d.'s (except the e.s.d. in the dihedral angle between two l.s. planes) are estimated using the full covariance matrix. The cell e.s.d.'s are taken into account individually in the estimation of e.s.d.'s in distances, angles and torsion angles; correlations between e.s.d.'s in cell parameters are only used when they are defined by crystal symmetry. An approximate (isotropic) treatment of cell e.s.d.'s is used for estimating e.s.d.'s involving l.s. planes.
Refinement. Refinement of *F*^2^ against ALL reflections. The weighted *R*-factor *wR* and goodness of fit *S* are based on *F*^2^, conventional *R*-factors *R* are based on *F*, with *F* set to zero for negative *F*^2^. The threshold expression of *F*^2^ > σ(*F*^2^) is used only for calculating *R*-factors(gt) *etc*. and is not relevant to the choice of reflections for refinement. *R*-factors based on *F*^2^ are statistically about twice as large as those based on *F*, and *R*- factors based on ALL data will be even larger.

### Fractional atomic coordinates and isotropic or equivalent isotropic displacement parameters (Å^2^)

**Table:**

	*x*	*y*	*z*	*U*_iso_*/*U*_eq_	
Si1	0.42728 (3)	0.37257 (3)	0.49618 (3)	0.02364 (14)	
Si2	0.41400 (3)	0.57131 (3)	0.44096 (3)	0.02422 (14)	
O1	0.42038 (7)	0.45998 (8)	0.44053 (6)	0.0257 (3)	
O2	0.49862 (7)	0.38739 (8)	0.55394 (6)	0.0272 (3)	
C1	0.35711 (12)	0.60943 (14)	0.52232 (11)	0.0382 (5)	
H1A	0.3064	0.5798	0.5215	0.057*	
H1B	0.3505	0.6758	0.5203	0.057*	
H1C	0.3843	0.5928	0.5682	0.057*	
C2	0.37276 (12)	0.60687 (14)	0.35079 (11)	0.0367 (5)	
H2A	0.4062	0.5858	0.3103	0.055*	
H2B	0.3689	0.6736	0.3492	0.055*	
H2C	0.3213	0.5801	0.3450	0.055*	
C11	0.45192 (10)	0.27253 (12)	0.43560 (9)	0.0243 (4)	
C21	0.46918 (11)	0.28754 (13)	0.36038 (10)	0.0298 (4)	
H21	0.4646	0.3475	0.3408	0.036*	
C31	0.49264 (11)	0.21768 (13)	0.31368 (10)	0.0332 (5)	
H31	0.5034	0.2298	0.2628	0.040*	
C41	0.50025 (12)	0.13027 (13)	0.34161 (11)	0.0329 (5)	
H41	0.5165	0.0820	0.3100	0.039*	
C51	0.48408 (11)	0.11334 (13)	0.41570 (10)	0.0305 (4)	
H51	0.4901	0.0533	0.4347	0.037*	
C61	0.45917 (10)	0.18265 (12)	0.46293 (10)	0.0240 (4)	
C71	0.43948 (10)	0.15854 (11)	0.54194 (10)	0.0252 (4)	
C81	0.36294 (11)	0.14336 (12)	0.56199 (10)	0.0307 (4)	
H81	0.3233	0.1526	0.5263	0.037*	
C91	0.34381 (12)	0.11508 (13)	0.63306 (11)	0.0347 (5)	
H91	0.2914	0.1045	0.6457	0.042*	
C101	0.40081 (13)	0.10219 (13)	0.68579 (11)	0.0358 (5)	
H101	0.3877	0.0827	0.7346	0.043*	
C111	0.47717 (12)	0.11785 (13)	0.66701 (10)	0.0342 (5)	
H111	0.5165	0.1096	0.7032	0.041*	
C121	0.49649 (11)	0.14550 (12)	0.59550 (10)	0.0300 (4)	
H121	0.5490	0.1556	0.5830	0.036*	
C12	0.33957 (11)	0.36475 (12)	0.55627 (10)	0.0264 (4)	
C22	0.35201 (12)	0.35934 (12)	0.63354 (10)	0.0319 (5)	
H22	0.4034	0.3554	0.6517	0.038*	
C32	0.29145 (13)	0.35950 (13)	0.68412 (11)	0.0386 (5)	
H32	0.3015	0.3561	0.7360	0.046*	
C42	0.21658 (13)	0.36468 (15)	0.65844 (12)	0.0433 (5)	
H42	0.1749	0.3641	0.6927	0.052*	
C52	0.20213 (12)	0.37074 (14)	0.58287 (11)	0.0388 (5)	
H52	0.1504	0.3745	0.5658	0.047*	
C62	0.26242 (11)	0.37139 (12)	0.53110 (10)	0.0298 (4)	
C72	0.24184 (11)	0.38291 (13)	0.45090 (11)	0.0299 (4)	
C82	0.26624 (11)	0.32206 (13)	0.39587 (10)	0.0328 (5)	
H82	0.2966	0.2707	0.4094	0.039*	
C92	0.24684 (12)	0.33543 (15)	0.32168 (11)	0.0392 (5)	
H92	0.2643	0.2936	0.2850	0.047*	
C102	0.20206 (12)	0.40966 (15)	0.30091 (11)	0.0399 (5)	
H102	0.1891	0.4190	0.2501	0.048*	
C112	0.17661 (12)	0.46966 (15)	0.35464 (11)	0.0392 (5)	
H112	0.1457	0.5205	0.3409	0.047*	
C122	0.19584 (11)	0.45615 (14)	0.42866 (11)	0.0346 (5)	
H122	0.1773	0.4977	0.4651	0.042*	

### Atomic displacement parameters (Å^2^)

**Table:**

	*U*^11^	*U*^22^	*U*^33^	*U*^12^	*U*^13^	*U*^23^
Si1	0.0279 (3)	0.0196 (3)	0.0235 (3)	−0.0010 (2)	0.0023 (2)	−0.0001 (2)
Si2	0.0271 (3)	0.0214 (3)	0.0241 (3)	0.0024 (2)	0.0005 (2)	0.0006 (2)
O1	0.0306 (7)	0.0207 (6)	0.0258 (6)	−0.0004 (5)	0.0028 (5)	0.0004 (5)
O2	0.0308 (7)	0.0240 (6)	0.0266 (7)	−0.0015 (5)	0.0002 (5)	0.0014 (5)
C1	0.0389 (12)	0.0349 (11)	0.0409 (12)	0.0038 (9)	0.0085 (9)	−0.0055 (9)
C2	0.0380 (12)	0.0349 (11)	0.0370 (11)	−0.0011 (9)	−0.0074 (9)	0.0067 (9)
C11	0.0237 (9)	0.0242 (9)	0.0251 (9)	−0.0016 (8)	−0.0005 (7)	−0.0011 (7)
C21	0.0359 (11)	0.0253 (10)	0.0282 (10)	0.0007 (8)	0.0037 (8)	0.0018 (8)
C31	0.0392 (12)	0.0334 (11)	0.0270 (10)	0.0006 (9)	0.0067 (8)	−0.0029 (8)
C41	0.0392 (12)	0.0278 (10)	0.0317 (10)	0.0028 (9)	0.0079 (9)	−0.0063 (8)
C51	0.0336 (11)	0.0220 (9)	0.0359 (11)	0.0030 (8)	0.0039 (9)	−0.0004 (8)
C61	0.0214 (9)	0.0242 (9)	0.0263 (9)	−0.0010 (7)	0.0000 (7)	0.0001 (7)
C71	0.0314 (10)	0.0155 (8)	0.0286 (10)	0.0006 (7)	0.0010 (8)	−0.0017 (7)
C81	0.0306 (11)	0.0295 (10)	0.0321 (10)	−0.0019 (8)	−0.0002 (8)	−0.0003 (8)
C91	0.0358 (12)	0.0348 (11)	0.0335 (11)	−0.0058 (9)	0.0071 (9)	0.0017 (9)
C101	0.0553 (14)	0.0257 (10)	0.0264 (10)	0.0006 (9)	0.0074 (9)	0.0003 (8)
C111	0.0422 (12)	0.0309 (11)	0.0295 (11)	0.0062 (9)	−0.0052 (9)	−0.0010 (8)
C121	0.0299 (10)	0.0267 (10)	0.0335 (11)	0.0039 (8)	0.0000 (8)	−0.0023 (8)
C12	0.0332 (10)	0.0194 (9)	0.0267 (9)	−0.0025 (8)	0.0051 (8)	−0.0018 (7)
C22	0.0390 (12)	0.0257 (10)	0.0309 (10)	−0.0029 (9)	0.0045 (9)	0.0001 (8)
C32	0.0524 (14)	0.0356 (12)	0.0278 (10)	−0.0059 (10)	0.0092 (10)	−0.0010 (9)
C42	0.0449 (14)	0.0489 (13)	0.0361 (12)	−0.0058 (11)	0.0183 (10)	−0.0042 (10)
C52	0.0325 (12)	0.0449 (13)	0.0390 (12)	−0.0043 (9)	0.0087 (9)	−0.0041 (10)
C62	0.0349 (11)	0.0239 (10)	0.0304 (10)	−0.0051 (8)	0.0064 (8)	−0.0041 (8)
C72	0.0239 (10)	0.0297 (10)	0.0360 (10)	−0.0065 (8)	0.0053 (8)	−0.0033 (8)
C82	0.0298 (11)	0.0332 (11)	0.0354 (11)	−0.0032 (9)	0.0027 (8)	−0.0048 (9)
C92	0.0349 (12)	0.0465 (13)	0.0361 (11)	−0.0050 (10)	0.0043 (9)	−0.0096 (10)
C102	0.0331 (12)	0.0549 (14)	0.0318 (11)	−0.0042 (10)	0.0011 (9)	0.0018 (10)
C112	0.0297 (11)	0.0446 (13)	0.0432 (12)	0.0014 (9)	0.0020 (9)	0.0052 (10)
C122	0.0282 (11)	0.0372 (11)	0.0386 (11)	−0.0029 (9)	0.0065 (9)	−0.0047 (9)

### Geometric parameters (Å, °)

**Table:**

Si1—O1	1.6287 (12)	C91—C101	1.382 (3)
Si1—O2	1.6290 (13)	C91—H91	0.9500
Si1—C12	1.8684 (19)	C101—C111	1.386 (3)
Si1—C11	1.8746 (17)	C101—H101	0.9500
Si2—O2^i^	1.6342 (13)	C111—C121	1.387 (3)
Si2—O1	1.6347 (12)	C111—H111	0.9500
Si2—C2	1.8452 (19)	C121—H121	0.9500
Si2—C1	1.8494 (19)	C12—C22	1.407 (3)
O2—Si2^i^	1.6342 (13)	C12—C62	1.415 (3)
C1—H1A	0.9800	C22—C32	1.388 (3)
C1—H1B	0.9800	C22—H22	0.9500
C1—H1C	0.9800	C32—C42	1.380 (3)
C2—H2A	0.9800	C32—H32	0.9500
C2—H2B	0.9800	C42—C52	1.383 (3)
C2—H2C	0.9800	C42—H42	0.9500
C11—C21	1.401 (2)	C52—C62	1.399 (3)
C11—C61	1.411 (2)	C52—H52	0.9500
C21—C31	1.384 (3)	C62—C72	1.493 (3)
C21—H21	0.9500	C72—C122	1.395 (3)
C31—C41	1.381 (3)	C72—C82	1.396 (3)
C31—H31	0.9500	C82—C92	1.388 (3)
C41—C51	1.382 (3)	C82—H82	0.9500
C41—H41	0.9500	C92—C102	1.387 (3)
C51—C61	1.392 (2)	C92—H92	0.9500
C51—H51	0.9500	C102—C112	1.378 (3)
C61—C71	1.501 (2)	C102—H102	0.9500
C71—C121	1.393 (3)	C112—C122	1.385 (3)
C71—C81	1.393 (3)	C112—H112	0.9500
C81—C91	1.382 (3)	C122—H122	0.9500
C81—H81	0.9500		
			
O1—Si1—O2	109.98 (7)	C71—C81—H81	119.5
O1—Si1—C12	110.05 (7)	C101—C91—C81	120.13 (19)
O2—Si1—C12	105.00 (8)	C101—C91—H91	119.9
O1—Si1—C11	105.97 (7)	C81—C91—H91	119.9
O2—Si1—C11	107.49 (7)	C91—C101—C111	119.62 (18)
C12—Si1—C11	118.22 (8)	C91—C101—H101	120.2
O2^i^—Si2—O1	107.88 (7)	C111—C101—H101	120.2
O2^i^—Si2—C2	107.71 (8)	C101—C111—C121	120.29 (18)
O1—Si2—C2	107.68 (8)	C101—C111—H111	119.9
O2^i^—Si2—C1	109.78 (8)	C121—C111—H111	119.9
O1—Si2—C1	109.94 (8)	C111—C121—C71	120.53 (18)
C2—Si2—C1	113.66 (10)	C111—C121—H121	119.7
Si1—O1—Si2	141.85 (8)	C71—C121—H121	119.7
Si1—O2—Si2^i^	142.12 (8)	C22—C12—C62	117.63 (17)
Si2—C1—H1A	109.5	C22—C12—Si1	116.64 (14)
Si2—C1—H1B	109.5	C62—C12—Si1	125.51 (14)
H1A—C1—H1B	109.5	C32—C22—C12	121.96 (19)
Si2—C1—H1C	109.5	C32—C22—H22	119.0
H1A—C1—H1C	109.5	C12—C22—H22	119.0
H1B—C1—H1C	109.5	C42—C32—C22	119.55 (19)
Si2—C2—H2A	109.5	C42—C32—H32	120.2
Si2—C2—H2B	109.5	C22—C32—H32	120.2
H2A—C2—H2B	109.5	C32—C42—C52	120.13 (19)
Si2—C2—H2C	109.5	C32—C42—H42	119.9
H2A—C2—H2C	109.5	C52—C42—H42	119.9
H2B—C2—H2C	109.5	C42—C52—C62	121.1 (2)
C21—C11—C61	117.57 (16)	C42—C52—H52	119.4
C21—C11—Si1	119.05 (13)	C62—C52—H52	119.4
C61—C11—Si1	123.23 (13)	C52—C62—C12	119.58 (18)
C31—C21—C11	122.07 (17)	C52—C62—C72	117.58 (18)
C31—C21—H21	119.0	C12—C62—C72	122.79 (16)
C11—C21—H21	119.0	C122—C72—C82	117.48 (18)
C41—C31—C21	119.56 (17)	C122—C72—C62	119.97 (17)
C41—C31—H31	120.2	C82—C72—C62	122.54 (17)
C21—C31—H31	120.2	C92—C82—C72	121.05 (19)
C31—C41—C51	119.77 (17)	C92—C82—H82	119.5
C31—C41—H41	120.1	C72—C82—H82	119.5
C51—C41—H41	120.1	C102—C92—C82	120.29 (19)
C41—C51—C61	121.25 (17)	C102—C92—H92	119.9
C41—C51—H51	119.4	C82—C92—H92	119.9
C61—C51—H51	119.4	C112—C102—C92	119.40 (19)
C51—C61—C11	119.77 (16)	C112—C102—H102	120.3
C51—C61—C71	118.35 (16)	C92—C102—H102	120.3
C11—C61—C71	121.87 (15)	C102—C112—C122	120.3 (2)
C121—C71—C81	118.42 (17)	C102—C112—H112	119.9
C121—C71—C61	121.58 (16)	C122—C112—H112	119.9
C81—C71—C61	119.90 (16)	C112—C122—C72	121.49 (19)
C91—C81—C71	121.01 (18)	C112—C122—H122	119.3
C91—C81—H81	119.5	C72—C122—H122	119.3
			
O1—Si2—O2^i^—Si1^i^	−74.36 (14)	C81—C91—C101—C111	0.1 (3)
O2—Si1—O1—Si2	−52.51 (15)	C91—C101—C111—C121	−0.6 (3)
C12—Si1—O1—Si2	62.69 (15)	C101—C111—C121—C71	0.5 (3)
C11—Si1—O1—Si2	−168.40 (12)	C81—C71—C121—C111	0.1 (3)
O2^i^—Si2—O1—Si1	84.84 (14)	C61—C71—C121—C111	−176.07 (16)
C2—Si2—O1—Si1	−159.17 (13)	O1—Si1—C12—C22	−127.17 (14)
C1—Si2—O1—Si1	−34.85 (16)	O2—Si1—C12—C22	−8.85 (15)
O1—Si1—O2—Si2^i^	−37.42 (15)	C11—Si1—C12—C22	110.94 (14)
C12—Si1—O2—Si2^i^	−155.78 (13)	O1—Si1—C12—C62	47.32 (17)
C11—Si1—O2—Si2^i^	77.52 (14)	O2—Si1—C12—C62	165.63 (15)
O1—Si1—C11—C21	7.60 (16)	C11—Si1—C12—C62	−74.57 (17)
O2—Si1—C11—C21	−109.97 (15)	C62—C12—C22—C32	0.6 (3)
C12—Si1—C11—C21	131.54 (15)	Si1—C12—C22—C32	175.55 (14)
O1—Si1—C11—C61	−176.83 (14)	C12—C22—C32—C42	0.3 (3)
O2—Si1—C11—C61	65.60 (16)	C22—C32—C42—C52	−0.8 (3)
C12—Si1—C11—C61	−52.89 (18)	C32—C42—C52—C62	0.3 (3)
C61—C11—C21—C31	−0.1 (3)	C42—C52—C62—C12	0.7 (3)
Si1—C11—C21—C31	175.77 (15)	C42—C52—C62—C72	−176.95 (19)
C11—C21—C31—C41	−0.7 (3)	C22—C12—C62—C52	−1.1 (3)
C21—C31—C41—C51	0.3 (3)	Si1—C12—C62—C52	−175.54 (14)
C31—C41—C51—C61	0.8 (3)	C22—C12—C62—C72	176.41 (17)
C41—C51—C61—C11	−1.6 (3)	Si1—C12—C62—C72	2.0 (3)
C41—C51—C61—C71	177.46 (18)	C52—C62—C72—C122	52.9 (2)
C21—C11—C61—C51	1.1 (3)	C12—C62—C72—C122	−124.6 (2)
Si1—C11—C61—C51	−174.49 (14)	C52—C62—C72—C82	−126.4 (2)
C21—C11—C61—C71	−177.85 (16)	C12—C62—C72—C82	56.1 (3)
Si1—C11—C61—C71	6.5 (2)	C122—C72—C82—C92	1.5 (3)
C51—C61—C71—C121	78.6 (2)	C62—C72—C82—C92	−179.20 (18)
C11—C61—C71—C121	−102.4 (2)	C72—C82—C92—C102	−0.5 (3)
C51—C61—C71—C81	−97.5 (2)	C82—C92—C102—C112	−0.4 (3)
C11—C61—C71—C81	81.5 (2)	C92—C102—C112—C122	0.3 (3)
C121—C71—C81—C91	−0.7 (3)	C102—C112—C122—C72	0.8 (3)
C61—C71—C81—C91	175.58 (17)	C82—C72—C122—C112	−1.7 (3)
C71—C81—C91—C101	0.6 (3)	C62—C72—C122—C112	179.02 (17)

Symmetry codes: (i) −*x*+1, −*y*+1, −*z*+1.
